# Pellagra

**Published:** 1846-03

**Authors:** 


*Pellagra.*—According to the report of the commission appointed to examine into the causes of this epidemic, its hereditary nature is clearly ascertained, and also the influence of insolation in producing the cutaneous erythema. The effects of bad and inefficient diet are equally well marked.—*Ibid.*

